# Three-Dimensional Digital Superimposition of Orthodontic Bracket Position by Using a Computer-Aided Transfer Jig System: An Accuracy Analysis

**DOI:** 10.3390/s21175911

**Published:** 2021-09-02

**Authors:** Jae-Hyun Park, Jin-Young Choi, Song Hee Oh, Seong-Hun Kim

**Affiliations:** 1 Department of Orthodontics, Graduate School of Dentistry, Kyung Hee University, Seoul 02447, Korea; cbx3748@naver.com (J.-H.P.); joyful.ortho@gmail.com (J.-Y.C.); 2 Department of Oral and Maxillofacial Radiology, Graduate School of Dentistry, Kyung Hee University, Seoul 02447, Korea; ohbbang50@gmail.com

**Keywords:** one-body transfer jig, indirect bonding, intraoral scanner, model scanner, 3D printing, best-fit method

## Abstract

Accurate bracket placement is essential for successful orthodontic treatment. An indirect bracket bonding system (IDBS) has been developed to ensure proper bracket positioning with three-dimensional computer-aided transfer jigs. The purpose of this study was to investigate the accuracy of bracket positioning by a one-body transfer jig according to the tooth type and presence/absence of a resin base. In total, 506 teeth from 20 orthodontic patients were included in this study. After initial dental models were scanned, virtual setup and bracket positioning procedures were performed with 3D software. Transfer jigs and RP models were fabricated with a 3D printer, and brackets were bonded to the RP model with or without resin base fabrication. The best-fit method of 3D digital superimposition was used to evaluate the lineal and angular accuracy of the actual bracket position compared to a virtual bracket position. Although all the measurements showed significant differences in position, they were clinically acceptable. Regarding the tooth types, premolars and molars showed higher accuracy than anterior teeth. The presence or absence of a resin base did not consistently affect the accuracy. In conclusion, the proper application of IDBS should be performed considering the errors, and resin base fabrication might not be essential in ensuring high-accuracy IDBS.

## 1. Introduction

Accurate placement of orthodontic brackets is one of the most important phases in comprehensive orthodontic treatment [1] in order to ensure the ideal occlusion described by Andrew’s six key principles [2,3]. Improper bracket positioning may result in unwanted tooth movement, such as unplanned torque, rotation, and extrusion/intrusion of teeth [4]. As the extraoral bracket position (i.e., indirect bonding system, IDBS) has been suggested to be as accurate as the intraoral direct bracket bonding by some authors, both direct and indirect bonding systems have been used to achieve the best orthodontic treatment results [5,6]. In addition to the accuracy, the reproducibility, reduced chair time, and lower risk of saliva contamination are considered advantages of IDBS compared to the direct bracket bonding system [7,8,9,10,11].

Since the first concept of IDBS was developed in 1972 by Silverman et al. [12], the technique has been upgraded with the development of bonding materials and transfer systems [13,14]. Before digital workflows were introduced into the IDBS, all the processes were performed manually, which required multiple time-consuming steps [15]. Digital software made the precise bracket placement possible with reduced lab time. After the brackets are placed accurately on the digital models, transferring them to the exact locations where the brackets are intended to be placed on the real teeth of the patient is important. A number of transfer tools have been used. A thermoformed transfer tray can be used in a simple procedure [15], but the tray material, a flexible sheet, is prone to deformation in the removal process, so that it is difficult to reuse. High flexibility may also affect the intraoral placement of the tray, which requires firm and precise sitting. Silicone materials and the combination of silicone materials with thermoplastic sheets have been also introduced [16]. Castilla et al. suggested that the accuracy of the bracket position of a silicone tray was similar to that of a thermoformed tray, but the consistency of the bracket position was higher when the silicone tray was used [17]. More recently, computer-aided design/computer-aided manufacturing (CAD/CAM) transfer trays have been introduced [18], and studies of its accuracy have been conducted. It was suggested that the CAD/CAM transfer tray shows clinically acceptable results and is comparable to a silicone tray [19]. Xue et al. also evaluated the accuracy of a newly designed bracket transfer device using CAD/CAM technology, and most of the measurements were within the clinically acceptable range, except for some brackets with torque deviation [20].

**Figure 1:**
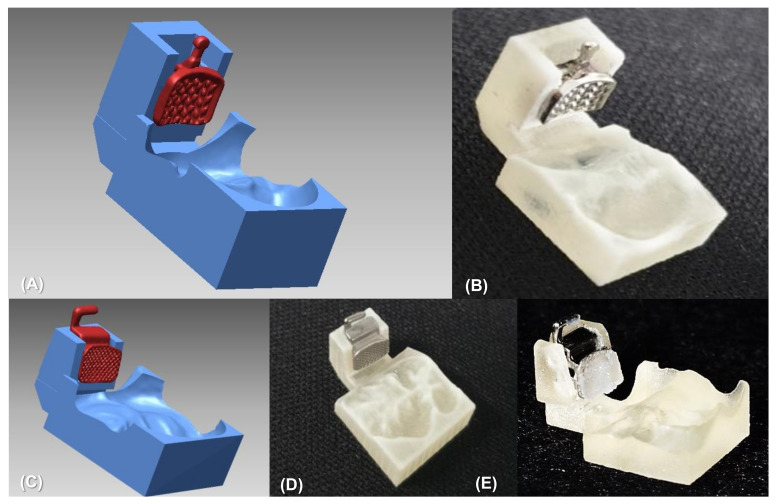
One-body transfer jig system based on CAD/CAM technology for indirect bonding. (**A**) An example of a transfer jig design for a premolar. (**B**) Manufactured transfer jig by three-dimensional printing technology. (**C**) Virtual design of transfer jig for a molar. (**D**) Bracket transfer jig precisely implemented with three-dimensional printing technology. (**E**) A customized resin base was formed through additional laboratory procedures.

**Figure 2:**
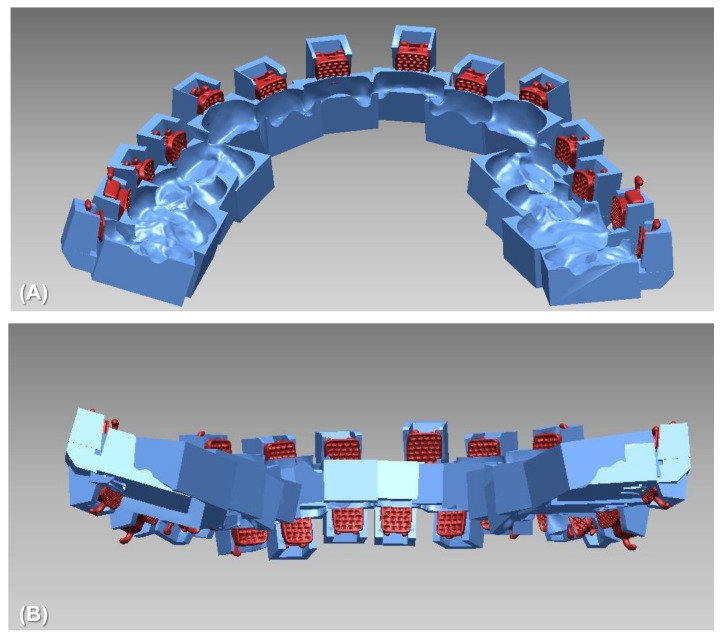
Examples of one-body jig system. (**A**) For maxillary dentition, (**B**) For whole dentition.

As one of the CAD/CAM transfer jig systems, a two-body transfer jig was developed and used for lingual orthodontic treatment [21,22]. The two-body jig has a part that holds the bracket and teeth separately, and the two parts are finally connected to perform a bracket bonding. However, there are many transfer errors because the bracket position needs to be guided by inserting a small sectional wire into the bracket slot. In order to overcome the limitations of the two-body transfer jig, a newly designed one-body transfer jig has been manufactured. Its contours adapt to all of the labial structures of the bracket, as well as the occlusal surface or incisal edge of the teeth, in one unit (Figure 1 and Figure 2). Considering its structure, the one-body transfer jig is expected to be more precise than the two-body transfer jig in placing the brackets in the planned position.

As shown in Figure 1E, the resin base is usually pre-fabricated and bonded to the bracket base due to the gap between the bracket base and the surface of the teeth. The resin base is essential for the conventional IDBS, and it contributes to the stability of bracket transfer [21,22]. However, the fabrication of the custom resin base requires an additional laboratory procedure, and the bond strength is lowered when the resin base is aged for a long period of time before being bonded to the teeth [23]. Because the average force for IDBS is greater than the force for direct bonding, showing a range from 150 to 780 g [24], there might be the possibility of differences in the bracket position according to the presence or absence of a resin base. The effect of the resin base on the accuracy of bracket positioning in IDBS has been evaluated, but only anterior dentition was included in the study [25].

Therefore, the purpose of this study was to evaluate the accuracy of the actual bracket position using a new type of one-body bracket transfer jig, according to the tooth type (i.e., anterior teeth, premolars, and molars) and presence/absence of a resin base.

## 2. Materials and Methods

### 2.1. Subjects

This study was approved by the Institutional Review Board of Kyung Hee University (IRB No. KH-DT19025), following the tenets of the Declaration of Helsinki. Permanent teeth of 20 patients who visited Kyung Hee University Dental Hospital for orthodontic treatment from March 2018 to February 2019 were selected for this in vitro study. Thirteen of them were female, and the remaining 7 subjects were male. Their age ranged from 13 to 48 years, and the average age was 22.3 years. The inclusion criteria for this study were: (1) teeth planned to be bonded with fixed orthodontic appliances using an indirect bonding system, (2) teeth in permanent dentition, (3) intact natural teeth without any defects or restorations, and (4) teeth to which the transfer jigs could be adapted in the planned position. A total of 506 teeth met the inclusion criteria and were selected for the measurements.

### 2.2. Study Procedure

#### 2.2.1. Fabrication of Transfer Jigs

**Figure 3:**
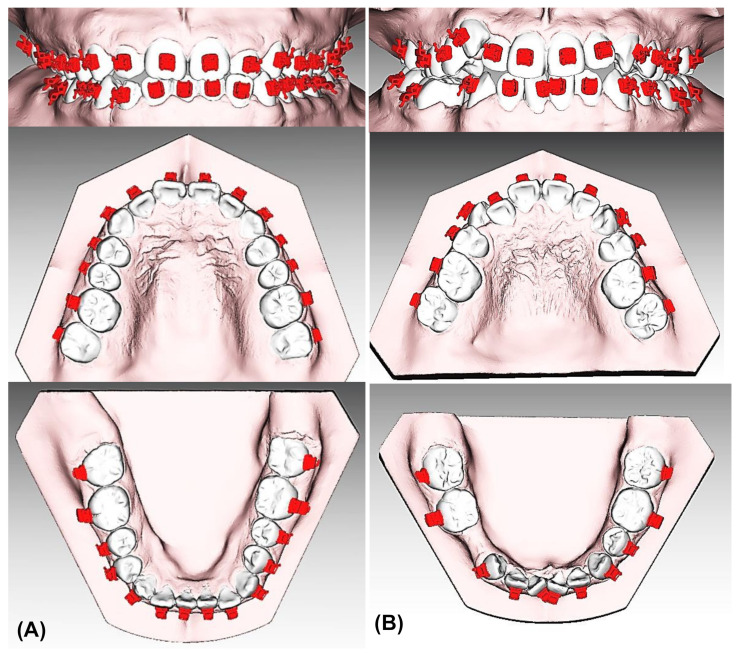
On the digital interface, Bioquick brackets were positioned virtually to the individual tooth surface using 3Txer software (CENOS co, Indeokwon, Gyeonggido, Korea). (**A**) Noncrowded case, (**B**) Crowded case.

**Figure 4:**
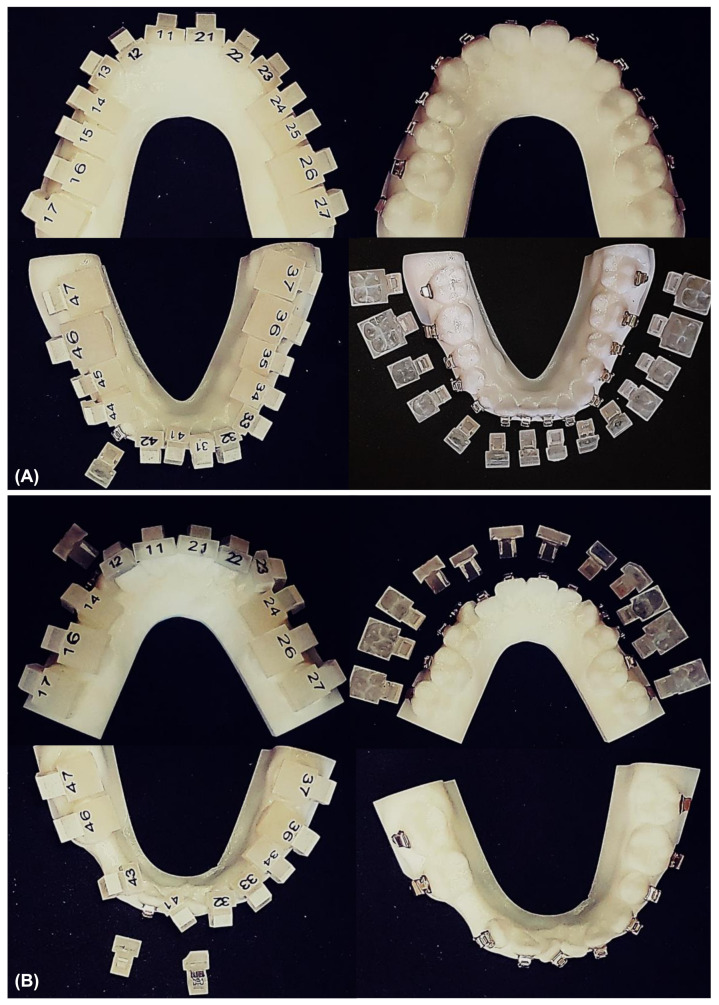
One-body transfer jig fabricated by CAD/CAM technology with rapid prototyping (RP) models. (**A**) Brackets transferred to the noncrowded model by indirect bonding procedure using the jig system, (**B**) Brackets transferred to the crowded model by indirect bonding procedure using the jig system.

**Figure 5:**
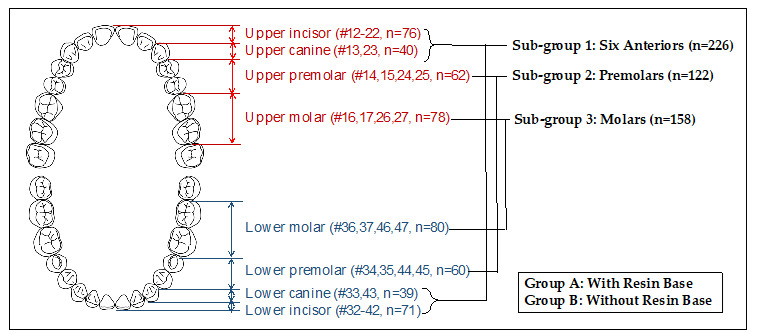
Schematic diagram of the experimental group. All subjects were classified into two groups depending on the presence or absence of a resin base (Group A: with resin base, Group B: without resin base). In addition, sub-groups were organized by tooth type. Sub-group 1 had six anterior teeth, a total of 226 subjects. Sub-groups 2 and 3 were the premolar and molar teeth, respectively, with a total of 112 and 158 subjects.

Initial dental models of the patients were scanned with a laser scanner (Medit T500, Medit Corp., Seoul, Korea) and converted to stereolithography (STL) files. Each tooth on the digital models was then separated, and virtual setup procedures were performed according to the treatment plan. Virtual orthodontic arch wires were brought onto the aligned teeth surfaces, and the orthodontic brackets were positioned with the aid of orthodontic archwires. Virtual setup and bracket positioning procedures were all performed with the 3Txer software (CENOS co, Indeokwon, Gyeonggido, Korea) (Figure 3). Bracket transfer jigs were designed with CAD/CAM software and were fabricated using a polyjet type 3D printer (Projet MJP 3600, 3D Systems Co., Rock Hill, SC, USA) (Figure 4). All the transfer jigs were fabricated twice and were divided into two groups. Orthodontic brackets with a resin base were classified as Group A, and the brackets without a resin base were classified as Group B (Figure 5). The self-ligation orthodontic metal brackets (0.022-in BioQuick^®^, Forestadent, Pforzheim, Germany) of Tweemac prescription [26] from central incisors to the second premolars and double and single tubes (3M Unitek, Monrovia, CA, USA) for first and second molars were inserted into the fabricated transfer jigs. Then, the resin base was fabricated and added to the base of the brackets in Group A with the following method [21,22,27]. Initial rapid prototyping (RP) models were printed using scanned data. Separating agents were applied to the tooth surface of the RP model, and the bracket base was washed and sand-blasted. After assembling the brackets and the transfer jigs, a bonding agent (Transbond™ XT Primer, 3M Unitek, Monrovia, CA, USA) and resin adhesive (Transbond™ XT Light Cure Adhesive, 3M Unitek, Monrovia, CA, USA) were applied sequentially to the bracket base. The assembly was placed on the initial RP model, and remnant resin was removed. Then, the remaining resin was light-cured using a light curing device (VALO, Ultradent, South Jordan, UT, USA).

#### 2.2.2. Indirect Bonding Procedures

One orthodontist (J.H.P.) bonded orthodontic brackets to the teeth of the RP model using transfer jigs. The surface of the RP model was treated with the bonding agent (Transbond™ XT Primer, 3M Unitek, Monrovia, CA, USA), and the brackets were bonded with the resin adhesive (Transbond™ XT Light Cure Adhesive, 3M Unitek, Monrovia, CA, USA). Although bonding agents and resin adhesive are bonding materials used for natural teeth, not for RP models, they were used to bond the brackets on the RP model in this study to reflect the clinical situation. The resin adhesive was cured with a light curing device (VALO, Ultradent, South Jordan, UT, USA). Then, the transfer jigs were separated from the orthodontic brackets bonded onto the surface of the RP model. After removing the jigs, RP models with the bonded brackets were scanned using an intraoral scanner (Trios 3, 3shape, Copenhagen, Denmark). The 3D information about each bracket position on the RP model was obtained through this process, and the images were converted to STL files to match with the scanned initial dental models, which were also converted to STL files.

### 2.3. Measurements

**Figure 6:**
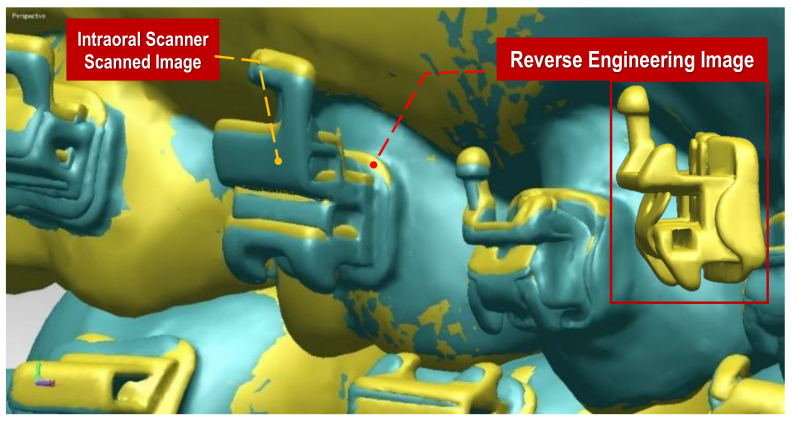
Three-dimensional digital superimposition (best-fit method) data. Combination between virtual model data with reverse engineering technique (yellow color) and intraoral scan data of post-transfer model (green color) using Rapidform software 2006 (INUS technology, Seoul, Korea).

**Figure 7:**
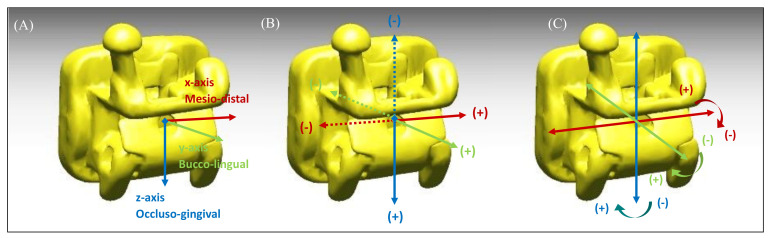
Three-dimensional coordinate system. The origin of the coordinate system was set to coincide with the center point of the bracket base. (**A**) The mesiodistal axis (*x*-axis) was to be parallel to the bracket slot (red color). The buccolingual direction (*y*-axis) was formed by drawing a normal line based on the lingual surface of the bracket slot (green color). The *z*-axis (occlusogingival direction) was determined to be perpendicular to the plane of the other two axes (blue color). (**B**) Measurement of linear bracket displacements. Positive values in each direction indicate mesial in the *x*-axis, occlusal in the *y*-axis, and buccal in the *z*-axis. (**C**) The values of angular discrepancy can be calculated between the coordinate vectors of the control group (virtual bracket position) and the experimental group (post-transfer bracket position) formed according to the preceding description. The rotation of the bracket with respect to the *x*-axis represents torque, the *y*-axis represents angulation, and the *z*-axis represents rotation. Positive values represent crown buccal torque, mesial root tip, and mesiobuccal rotation.

The virtual bracket position on the scanned initial dental models and the actual bracket position on the RP models were superimposed with a best-fit algorithm, and the differences were measured with Rapidform software 2006 (INUS technology, Seoul, Korea) by one orthodontist (J.H.P.) (Figure 6). For three-dimensional analyses, a linear coordinate system (x-, y-, and z-axes) was constructed for each bracket based on the midpoint of the bracket base (Figure 7). Three linear differences and three angular differences between them were measured in each tooth. The virtual bracket position was considered the baseline, and the differences in the actual bracket position from the baseline were recorded. To evaluate the reproducibility of the measurements, 30 teeth were randomly chosen, and all the measurements were measured by the same researcher after an interval of 2 weeks. Bland–Altman plots were used to assess the reproducibility of the measured values.

#### 2.3.1. Linear Measurements

- Mesiodistal direction (M-D): a linear measurement (mm) of a discrepancy along the *x*-axis. The discrepancy in the mesial direction was recorded as a positive value, and the discrepancy in the distal direction was recorded as a negative value.
- Buccolingual direction (B-L): a linear measurement (mm) of a discrepancy along the *y*-axis. The discrepancy in the buccal direction was recorded as a positive value, and the discrepancy in the lingual direction was recorded as a negative value.
- Occlusogingival direction (O-G): a linear measurement (mm) of a discrepancy along the *z*-axis. The discrepancy in the occlusal direction was recorded as a positive value, and the discrepancy in the apical direction was recorded as a negative value.

#### 2.3.2. Angular Measurements

- Torque (T): an angular measurement (°) of a discrepancy between the *y*-axis on the virtual model and the *y*-axis on the actual model, projected to the y–z plane of the virtual model. The torque discrepancy in the crown lingual direction was recorded as a positive value, and the torque discrepancy in the crown labial/buccal direction was recorded as a negative value.
- Angulation (A): an angular measurement (°) of a discrepancy between the *x*-axis on the virtual model and the *x*-axis on the actual model, projected to the x–z plane of the virtual model. The angulation discrepancy in the crown distal direction was recorded as a positive value, and the angulation discrepancy in the crown mesial direction was recorded as a negative value.
- Rotation (R): an angular measurement (°) of a discrepancy between the *y*-axis on the virtual model and the *y*-axis on the actual model, projected to the x–y plane of the virtual model. The rotation discrepancy in the distobuccal direction was recorded as a positive value, and the rotation discrepancy in the mesiobuccal direction was recorded as a negative value.

### 2.4. Statistical Analysis

All the statistical analyses were performed with SPSS 22.0 (IBM, Armonk, NY, USA). A normality test was conducted via the Kolmogorov–Smirov test, and it showed normal distribution for all the outcomes (*p* > 0.05). To verify the resultant similarity between the laser scanning procedure and intraoral scanning procedure, the same points on each scanned model were compared using a one-sample *t*-test, based on the verification value of 0.15 mm with 10% zone of equivalence. An equivalence test, the two one-sided *t*-test (TOST), showed no statistically significant differences between the two scanning procedures.

The differences in linear and angular measurements between virtual and actual bracket positions were evaluated with a one-sample *t*-test. Homogeneity of variances was confirmed for all the data by the Levene test (*p* > 0.05). After dividing samples into three sub-groups according to the position of teeth (sub-group A composed of incisors and canines; sub-group B composed of premolars; and sub-group C composed of molars), comparison of differences among the sub-groups was evaluated with a one-way analysis of variance (ANOVA) followed by *t*-test with Bonferroni correction (Figure 5). Intergroup comparison between groups A and B, which were divided according to the presence/absence of a resin base, in each sub-group was conducted with an independent *t*-test. Finally, a two-way analysis of covariance (ANCOVA) was used to verify the interactions of the two groups and three sub-groups with the pooling technique.

Furthermore, 0.5 mm of linear discrepancy and 2° of angular discrepancy were assumed to be a clinically acceptable range, based on the American Board of Orthodontics Objective Grading System (ABO OGS). Based on these criteria, the frequency of differences was examined with a one-tailed equivalence test, and the directional bias was also evaluated.

## 3. Results

### 3.1. Overall Differences in Bracket Position

**Figure 8:**
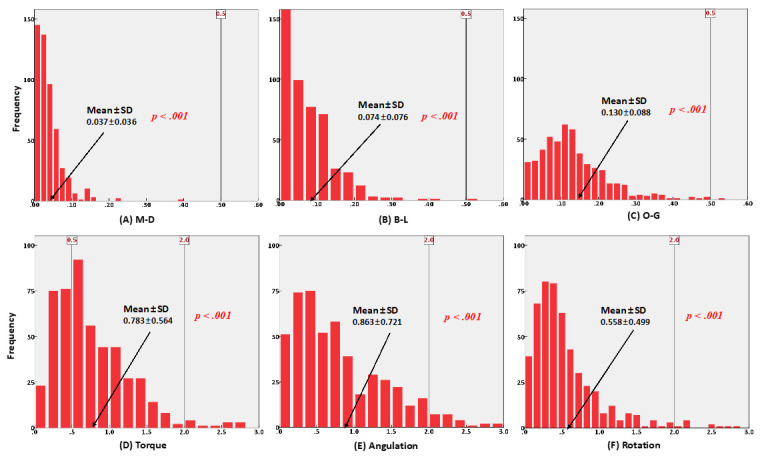
Histogram of frequencies for six measurements with one-tailed equivalence test in group A. (**A**–**C**) linear measurements, (**D**–**F**) angular measurements.

**Figure 9:**
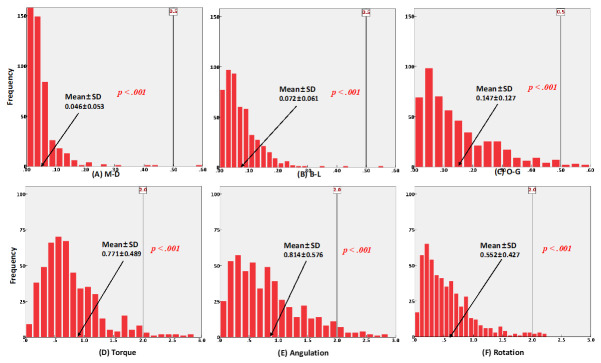
Histogram of frequencies for six measurements with one-tailed equivalence test in group B. (**A**–**C**) linear measurements, (**D**–**F**) angular measurements.

Table 1 shows the overall three-dimensional differences between the bracket positions on each tooth type in the virtual and actual models. All the measurements showed statistically significant differences in the actual bracket positions compared to the virtual bracket positions in all types of teeth. The results of the one-tailed equivalence test for linear measurements and angular measurements in groups A and B are shown in Figure 8 and Figure 9, respectively.

The actual bracket positions showed directional bias compared to the planned bracket positions in the virtual models. Table 2 shows the directional bias for each group. There were tendencies of the brackets to be bonded mesially and occlusally with buccal crown torque.

### 3.2. Differences in Bracket Position According to Tooth Type

Table 3 shows the results of comparing the bracket position differences among six anterior teeth, premolars, and molars. All the measurements showed statistically significant differences. Specific comparisons between six anterior teeth and premolars, between six anterior teeth and molars, and between premolars and molars in groups A and B are suggested in Table 4 and Table 5. While all the measurements were different between six anterior teeth and premolars, and between six anterior teeth and molars, except for an occlusogingival difference between six anterior teeth and molars, there were no differences in bracket position errors between premolars and molars, except for an occlusogingival difference, in group A (Table 4). The differences in bracket position between actual and virtual models were the largest in the six anterior teeth. Bracket position differences in group B were similar to the differences in group A. However, the mesiodistal difference between six anterior teeth and premolars, and the buccolingual difference between premolars and molars, did not show statistical significance, and the mesiodistal bracket position error was different between premolars and molars in group B (Table 5).

### 3.3. Differences in Bracket Position According to the Presence/Absence of Resin Base

The effect of resin bases on the bracket position differences is shown in Table 6. Mesiodistal, buccolingual, and angulation differences in the six anterior teeth were larger in group A than group B, and the occlusogingival differences in the six anterior teeth were larger in group B than group A. Brackets bonded on the premolars and molars showed more mesiodistal differences in group B than group A. All the other measurements showed no significant differences between group A and B.

### 3.4. Interactions between Tooth Type and Resin Base

**Figure 10:**
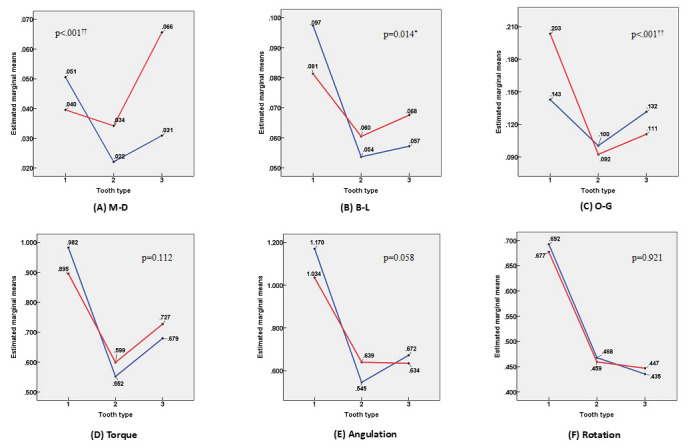
Profile plot for estimated marginal means of (**A**) M-D, (**B**) B-L, (**C**) O-G, (**D**) torque, (**E**) angulation, and (**F**) rotation. Tooth type 1, 2, and 3 represent six anterior teeth, premolars, and molars, respectively. The blue line represents the experimental group with resin base formed in advance, and the red line represents the group without resin base.

Despite the high accuracy of the superimposition between the initial model scan and intraoral scan data, superimposition error could occur in the preparation process and/or in the computer programs. This error was considered as an exogenous variable (covariate) and the interactions between independent variables were investigated using two-way ANCOVA [28]. Figure 10 shows that the interactions between tooth type and resin base occurred in the mesiodistal, buccolingual, and occlusogingival variables. In contrast, there were no interactions between tooth type and resin base in the torque, angulation, and rotation variables.

## 4. Discussion

With the aid of CAD/CAM systems, high-quality orthodontic treatment is expected to be achieved [29,30]. The accuracy of bracket transfer jigs has also increased as the technologies, especially CAD/CAM systems, have been developed. The overall accuracy of the one-body transfer jigs used in this study showed clinically acceptable results that were similar to previous studies. Although all the measurements were significantly different between the virtual bracket position on the computer software and the actual bonded position on the RP model, which is confirmed in Table 1, most measurements are considered to be sufficiently accurate based on the one-tailed equivalent test, as shown in Figure 8 and Figure 9. As mentioned above, 0.5 mm of linear discrepancy and 2° of angular discrepancy were assumed to be clinically acceptable values based on the ABO OGS. The vertical lines in Figure 8 and Figure 9 represent the ABO OGS criterion, and almost all measurements were within this criterion, regardless of whether the resin base was present.

In previous studies on the accuracy of CAD/CAM transfer jig systems, variables affecting the accuracy were not considered [20,31,32,33,34,35]. In this study, we sought to consider such factors in order to interpret the results in terms of their accuracy. An assumption of consistency between the scanned data of the initial model by the model scanner and the scanned data of the RP model by the intraoral scanner is required to analyze the results. It is assumed that there was no deviation in superimposition between them [36,37]. If so, these two scanned datasets are superimposed, and there should be no error between them. However, this is technically impossible in a computer program. In order to consider the effect of this discrepancy for all six measurements, the corresponding deviation in the direction of each coordinate system must be investigated. Since this is not practically possible, the average value was obtained for the positional difference between the two datasets on the superimposition program for each tooth. Then, this was controlled when statistically processed with two-way ANCOVA to determine the effect of the interaction between the independent variables. It was concluded that the superimposition process might have introduced an error into the linear measurements, which showed statistically significant differences.

Directional bias was also evaluated, as it could affect the accuracy of the bracket transfer jigs. Although a graph was not generated, the three main measurements that showed directional bias were M-D, O-G, and torque, based on the results shown in Table 2. In particular, 90.9% of the brackets in group A and 90.7% of the brackets in group B were positioned occlusally, which showed the highest directional bias. Due to the contours of tooth surfaces, torque is affected by the occlusogingival position of the bracket. Buccal crown torque tendencies might diminish the resultant torque factors from the occlusally positioned brackets, but this would not be guaranteed due to the inconsistency among the measurements. When the transfer jig is positioned, indirect bonding should be performed while applying a vertical force by hand in the direction of the long axis of the tooth from the occlusal surface [38]. The directional bias result, despite the proper application of finger pressure in the process of bonding in this study, suggests that firm and strong pressure would be required in the clinical situation.

Among the three independent variables considered in this study, the effect of the resin base was not noticeable, particularly in the molars. As shown in Table 6, only M-D showed statistically significant differences according to the resin base in sub-groups 2 and 3. This is meaningful because the posterior teeth are the region where the influence of the resin base could be identified. The effect of the resin base on each of the six dependent variables for the incisors and canines (sub-group 1) showed significant differences, except for torque and rotation, but there was no consistent tendency. This suggests that the results are mixed as the independent variable of tooth type interacts with the resin base. In the two-way ANCOVA results shown in Figure 10, we can see the interaction between tooth type and resin base in the controlled state of the covariate. In the graph, the *x*-axis represents 1 for anterior teeth, 2 for premolars, and 3 for molars depending on the tooth type. The *y*-axis is the estimated marginal mean value. The red line represents the case with no resin base and the blue line represents the case with the resin base. The slopes of the red and blue lines in each graph are different due to the interaction between tooth type and the resin base. According to this analysis, the interaction between resin base and tooth type affected the linear measurements (M-D, B-L, and O-G).

Interestingly, the differences between the virtual and actual bracket positions in the M-D and B-L direction, as well as angulation, in group A were larger than those in group B. Considering the purpose of fabricating a resin base, i.e., to ensure the accurate placement of the bracket, this result was contrary to the expected outcomes. It might be due to the possible errors arising during the laboratory process. The resin base is pre-fabricated on the dental model in advance, as described in the Methods section. This resin base is not printed with the CAD/CAM system, unlike the other components of the transfer jig; rather, the dental technicians construct it by hand. When the resin base is thicker due to the large gap between the bracket base and the tooth surface, the possibility of errors is increased. Based on the results in this study, we cannot conclude that fabricating a resin base is more advantageous than filling the gap with bonding materials only in the bonding process. Moreover, clinically acceptable accuracy was achieved with the one-body transfer jigs used in this study. Thus, by eliminating the prior fabrication of the resin base, it would be possible to decrease the effort, time, and cost required to construct the resin base in the laboratory.

There would be various regulatory factors in actual clinical practice in addition to the considered variables, as this was an in vitro study. For example, unexpected movement of tissues around the teeth, such as tongue or cheeks, or excessive salivation can interfere with the correct transferring of the bracket. In addition, discrepancies are inevitable in the many experimental processes, such as digital scanning, RP model fabrication, resin base formation, and possible computer program errors. In particular, the bracket cannot be scanned with a sufficient resolution to form a precise coordinate system in the digital intraoral scan data of the RP model. To compensate for this, digital information was generated by scanning the entire bracket using high-resolution computer tomography (CT) in advance [39]. Therefore, if these are not considered, bracket transfer errors may appear more severe than they truly are. As such, it was possible to evaluate the accuracy of the bracket transfer device in this study only by controlling the influence of exogenous variables that are likely to affect the result.

For this study, the groups were classified based on the presence or absence of a resin base, and the samples were divided into three sub-groups: anterior teeth, premolars, and molars. Different teeth even in the same sub-group have the potential to lead to differences in accuracy. For example, upper central incisors and lower central incisors are classified into the same sub-group, but they have different shapes and sizes, which might result in different measurements. Differences between the upper and lower teeth will be studied in future research.

## Figures and Tables

**Table 1:** Difference between the reference position and the actual bracket placement after indirect bonding for each experimental group.

Group	Tooth Type (n)	Mesiodistal (mm)		Buccolingual (mm)		Occlusogingival (mm)		Torque (°)		Angulation (°)		Rotation (°)	
		Mean ± SD	*p*	Mean ± SD	*p*	Mean ± SD	*p*	Mean ± SD	*p*	Mean ± SD	*p*	Mean ± SD	*p*
A	1 (226)	0.050 ±0.042	<0.001 ^††^	0.097 ±0.081	<0.001 ^††^	0.142 ±0.099	<0.001 ^††^	0.982 ±0.708	<0.001 ^††^	1.169 ±0.824	<0.001 ^††^	0.692 ±0.616	<0.001 ^††^
	2 (122)	0.022 ±0.026	<0.001 ^††^	0.053 ±0.070	<0.001 ^††^	0.100 ±0.055	<0.001 ^††^	0.551 ±0.299	<0.001 ^††^	0.545 ±0.442	<0.001 ^††^	0.467 ±0.396	<0.001 ^††^
	3 (158)	0.030 ±0.027	<0.001 ^††^	0.057 ±0.062	<0.001 ^††^	0.131 ±0.084	<0.001 ^††^	0.679 ±0.358	<0.001 ^††^	0.671 ±0.545	<0.001 ^††^	0.435 ±0.300	<0.001 ^††^
B	1 (226)	0.039 ±0.033	<0.001 ^††^	0.081 ±0.057	<0.001 ^††^	0.203 ±0.139	<0.001 ^††^	0.895 ±0.527	<0.001 ^††^	1.035 ±0.576	<0.001 ^††^	0.676 ±0.485	<0.001 ^††^
	2 (122)	0.034 ±0.030	<0.001 ^††^	0.060 ±0.071	<0.001 ^††^	0.092 ±0.085	<0.001 ^††^	0.598 ±0.414	<0.001 ^††^	0.639 ±0.488	<0.001 ^††^	0.459 ±0.342	<0.001 ^††^
	3 (158)	0.065 ±0.080	<0.001 ^††^	0.097 ±0.054	<0.001 ^††^	0.111 ±0.103	<0.001 ^††^	0.726 ±0.436	<0.001 ^††^	0.633 ±0.531	<0.001 ^††^	0.447 ±0.344	<0.001 ^††^

Mean, mean value of difference; SD, standard deviation. n is number of brackets used for analysis. *p* value was calculated using one-sample *t*-test. ^††^ *p* < 0.001. Tooth type 1, 2, and 3 represent six anterior teeth, premolars, and molars, respectively. Group A is the experimental group with resin base formed in advance, and group 2 is the group without resin base.

**Table 2:** Frequencies of directional bias for each experimental group.

Group	Tooth Type (n)	Mesiodistal (mm)		Buccolingual (mm)		Occlusogingival (mm)		Torque (°)		Angulation (°)		Rotation (°)	
		Mesial	Distal	Buccal	Lingual	Occlusal	Gingival	BCT	LCT	MRT	DRT	m-b	m-l
A	1 (226)	188	38	119	107	208	18	141	85	104	122	107	119
	2 (122)	62	60	61	61	107	15	70	52	62	60	63	59
	3 (158)	71	87	82	76	145	13	108	50	66	91	83	75
B	1 (226)	140	86	130	96	214	12	151	75	96	130	121	105
	2 (122)	81	41	62	60	105	17	84	38	72	50	54	68
	3 (158)	86	72	88	70	140	18	87	71	75	83	73	85

BCT, buccal crown torque; LCT, lingual crown torque; MRT, mesial root tip; DRT, distal root tip; m-b, mesiobuccal; m-l, mesiolingual. Tooth type 1, 2, and 3 represent six anterior teeth, premolars, and molars, respectively. Group A is the experimental group with resin base formed in advance, and group 2 is the group without resin base.

**Table 3:** Comparison according to the tooth type for each experimental group.

Group	Mesiodistal (mm)				Buccolingual (mm)				Occlusogingival (mm)			
	1 (226)	2 (122)	3 (158)	*p*	1 (226)	2 (122)	3 (158)	*p*	1 (226)	2 (122)	3 (158)	*p*
A	0.050 ±0.042	0.022 ±0.026	0.030 ±0.027	<0.001 ^††^	0.097 ±0.081	0.053 ±0.070	0.057 ±0.062	<0.001 ^††^	0.142 ±0.099	0.100 ±0.055	0.131 ±0.084	<0.001 ^††^
B	0.039 ±0.033	0.034 ±0.030	0.065 ±0.080	<0.001 ^††^	0.081 ±0.057	0.060 ±0.071	0.067 ±0.054	0.004 ^†^	0.203 ±0.139	0.092 ±0.085	0.111 ±0.103	<0.001 ^††^
**Group**	**Torque (°)**				**Angulation (°)**				**Rotation (°)**			
	**1** **(226)**	**2** **(122)**	**3** **(158)**	***p***	**1** **(226)**	**2** **(122)**	**3** **(158)**	***p***	**1** **(226)**	**2** **(122)**	**3** **(158)**	***p***
A	0.982 ±0.708	0.551 ±0.299	0.679 ±0.358	<0.001 ^††^	1.169 ±0.824	0.545 ±0.442	0.671 ±0.545	<0.001 ^††^	0.692 ±0.616	0.467 ±0.396	0.435 ±0.300	<0.001 ^††^
B	0.895 ±0.527	0.598 ±0.414	0.726 ±0.436	<0.001 ^††^	1.035 ±0.576	0.639 ±0.488	0.633 ±0.531	<0.001 ^††^	0.676 ±0.485	0.459 ±0.342	0.447 ±0.344	<0.001 ^††^

Values are expressed as mean ± standard deviation. One-way analysis of variance (ANOVA) test was performed. ^†^ *p* < 0.01; ^††^ *p* < 0.001. Tooth type 1, 2, and 3 represent six anterior teeth, premolars, and molars, respectively. Group A is the experimental group with resin base formed in advance, and group 2 is the group without resin base.

**Table 4:** Mutual comparison between six anterior teeth, premolars, and molars (group A).

Tooth Type (n)		Mesiodistal (mm)			Buccolingual (mm)			Occlusogingival (mm)		
		Mean Difference	SD	*p*	Mean Difference	SD	*p*	Mean Difference	SD	*p*
1 (226)	2 (122)	0.028	0.003	<0.001 ^††^	0.043	0.008	<0.001 ^††^	0.042	0.009	<0.001 ^††^
	3 (158)	0.019	0.003	<0.001 ^††^	0.040	0.007	<0.001 ^††^	0.011	0.008	0.611
2 (122)	3 (158)	−0.008	0.004	0.107	−0.003	0.008	>0.999	−0.031	0.010	0.008 ^†^
**Tooth Type** **(n)**		**Torque (°)**			**Angulation (°)**			**Rotation (°)**		
		**Mean Difference**	**SD**	***p***	**Mean Difference**	**SD**	***p***	**Mean Difference**	**SD**	***p***
1 (226)	2 (122)	0.430	0.060	<0.001 ^††^	0.624	0.074	<0.001 ^††^	0.224	0.054	<0.001 ^††^
	3 (158)	0.302	0.055	<0.001 ^††^	0.497	0.069	<0.001 ^††^	0.257	0.050	<0.001 ^††^
2 (122)	3 (158)	−0.127	0.064	0.146	−0.121	0.080	0.351	0.032	0.058	>0.999

Mean, mean value of difference; SD, standard deviation. n is number of brackets used for analysis. *p* value was calculated using *t*-test with Bonferroni correction. ^†^ *p* < 0.01; ^††^ *p* < 0.001. Tooth type 1, 2, and 3 represent six anterior teeth, premolars, and molars, respectively.

**Table 5:** Mutual comparison between six anterior teeth, premolars, and molars (group B).

Tooth Type (n)		Mesiodistal (mm)			Buccolingual (mm)			Occlusogingival (mm)		
		Mean Difference	SD	*p*	Mean Difference	SD	*p*	Mean Difference	SD	*p*
1 (226)	2 (122)	0.005	0.058	>0.999	0.021	0.006	0.006 ^†^	0.111	0.013	<0.001 ^††^
	3 (158)	−0.025	0.005	<0.001 ^††^	0.014	0.006	0.076	0.092	0.012	<0.001 ^††^
2 (122)	3 (158)	−0.031	0.006	<0.001 ^††^	−0.007	0.007	>0.999	−0.018	0.014	0.566
**Tooth Type** **(n)**		**Torque (°)**			**Angulation (°)**			**Rotation (°)**		
		**Mean Difference**	**SD**	***p***	**Mean Difference**	**SD**	***p***	**Mean Difference**	**SD**	***p***
1 (226)	2 (122)	0.296	0.053	<0.001 ^††^	0.396	0.060	<0.001 ^††^	0.216	0.046	<0.001 ^††^
	3 (158)	0.168	0.049	0.002 ^†^	0.402	0.056	<0.001 ^††^	0.229	0.042	<0.001 ^††^
2 (122)	3 (158)	−0.127	0.057	0.077	0.005	0.065	>0.999	0.012	0.049	>0.999

Mean, mean value of difference; SD, standard deviation. n is number of brackets used for analysis. *p* value was calculated using *t*-test with Bonferroni correction. ^†^ *p* < 0.01; ^††^ *p* < 0.001. Tooth type 1, 2, and 3 represent six anterior teeth, premolars, and molars, respectively.

**Table 6:** Intergroup comparison according to the presence or absence of resin base.

Tooth Type (n)	Mesiodistal (mm)			Buccolingual (mm)			Occlusogingival (mm)		
	A	B	*p*	A	B	*p*	A	B	*p*
	Mean ± SD	Mean ± SD		Mean ± SD	Mean ± SD		Mean ± SD	Mean ± SD	
1 (226)	0.050 ±0.042	0.039 ±0.033	0.003 ^†^	0.097 ±0.081	0.081 ±0.057	0.018 *	0.142 ±0.099	0.203 ±0.139	<0.001 ^††^
2 (122)	0.022 ±0.026	0.034 ±0.030	0.001 ^†^	0.053 ±0.070	0.060 ±0.071	0.459	0.100 ±0.055	0.092 ±0.085	0.390
3 (158)	0.030 ±0.027	0.065 ±0.080	<0.001 ^††^	0.057 ±0.062	0.067 ±0.054	0.119	0.131 ±0.084	0.111 ±0.103	0.053
**Tooth Type (n)**	**Torque (°)**			**Angulation (°)**			**Rotation (°)**		
	**A**	**B**	***p***	**A**	**B**	***p***	**A**	**B**	***p***
	**Mean ± SD**	**Mean ± SD**		**Mean ± SD**	**Mean ± SD**		**Mean ± SD**	**Mean ± SD**	
1 (226)	0.982 ±0.708	0.895 ±0.527	0.141	1.169 ±0.824	1.035 ±0.576	0.046 *	0.692 ±0.616	0.676 ±0.485	0.757
2 (122)	0.551 ±0.299	0.598 ±0.414	0.311	0.545 ±0.442	0.639 ±0.488	0.117	0.467 ±0.396	0.459 ±0.342	0.863
3 (158)	0.679 ±0.358	0.726 ±0.436	0.290	0.671 ±0.545	0.633 ±0.531	0.531	0.435 ±0.300	0.447 ±0.344	0.750

Mean, mean value of difference; SD, standard deviation. n is number of brackets used for analysis. Independent *t*-test was performed for calculating *p* value. * *p* < 0.05; ^†^ *p* < 0.01; ^††^ *p* < 0.001. Tooth type 1, 2, and 3 represent six anterior teeth, premolars, and molars, respectively. Group A is the experimental group with resin base formed in advance, and group 2 is the group without resin base.

## References

[1] Creekmore T.D., Kunik R.L. (1993). Straight wire: The next generation. Am. J. Orthod. Dentofac. Orthop..

[2] Andrews L.F. (1976). The straight-wire appliance. Explained and compared. J. Clin. Orthod..

[3] Andrews L.F. (1979). The straight-wire appliance. Br. J. Orthod..

[4] Nawrocka A., Lukomska-Szymanska M. (2020). The Indirect Bonding Technique in Orthodontics—A Narrative Literature Review. Materials.

[5] Deahl S.T., Salome N., Hatch J.P., Rugh J.D. (2007). Practice-based comparison of direct and indirect bonding. Am. J. Orthod. Dentofac. Orthop..

[6] Zachrisson B.U., Brobakken B.O. (1978). Clinical comparison of direct versus indirect bonding with different bracket types and adhesives. Am. J. Orthod..

[7] Aguirre M.J., King G.J., Waldron J.M. (1982). Assessment of bracket placement and bond strength when comparing direct bonding to indirect bonding techniques. Am. J. Orthod..

[8] Koo B.C., Chung C.H., Vanarsdall R.L. (1999). Comparison of the accuracy of bracket placement between direct and indirect bonding techniques. Am. J. Orthod. Dentofac. Orthop..

[9] Hodge T.M., Dhopatkar A.A., Rock W.P., Spary D.J. (2004). A randomized clinical trial comparing the accuracy of direct versus indirect bracket placement. J. Orthod..

[10] Shpack N., Geron S., Floris I., Davidovitch M., Brosh T., Vardimon A.D. (2007). Bracket placement in lingual vs. labial systems and direct vs. indirect bonding. Angle Orthod..

[11] Bozelli J.V., Bigliazzi R., Barbosa H.A., Ortolani C.L., Bertoz F.A., Faltin K. (2013). Comparative study on direct and indirect bracket bonding techniques regarding time length and bracket detachment. Dent. Press J. Orthod..

[12] Silverman E., Cohen M., Gianelly A.A., Dietz V.S. (1972). A universal direct bonding system for both metal and plastic brackets. Am. J. Orthod..

[13] Kalange J.T. (2004). Indirect bonding: A comprehensive review of the advantages. World J. Orthod..

[14] Thomas R.G. (1979). Indirect bonding: Simplicity in action. J. Clin. Orthod..

[15] Layman B. (2019). Digital Bracket Placement for Indirect Bonding. J. Clin. Orthod..

[16] Möhlhenrich S.C., Alexandridis C., Peters F., Kniha K., Modabber A., Danesh G., Fritz U. (2020). Three-dimensional evaluation of bracket placement accuracy and excess bonding adhesive depending on indirect bonding technique and bracket geometry: An in-vitro study. Head Face Med..

[17] Castilla A.E., Crowe J.J., Moses J.R., Wang M., Ferracane J.L., Covell D.A. (2014). Measurement and comparison of bracket transfer accuracy of five indirect bonding techniques. Angle Orthod..

[18] Seo H. (2016). Accuracy of Indirect Bracket Bonding Via Virtual Setup and 3D Printing. Master’s Thesis.

[19] Pottier T., Brient A., Turpin Y.L., Chauvel B., Meuric V., Sorel O., Brezulier D. (2020). Accuracy evaluation of bracket repositioning by indirect bonding: Hard acrylic CAD/CAM versus soft one-layer silicone trays, an in vitro study. Clin. Oral Investig..

[20] Xue C., Xu H., Guo Y., Xu L., Dhami Y., Wang H., Liu Z., Ma J., Bai D. (2020). Accurate bracket placement using a computer-aided design and computer-aided manufacturing-guided bonding device: An in vivo study. Am. J. Orthod. Dentofac. Orthop..

[21] Fillion D. (2010). Clinical advantages of the Orapix-straight wire lingual technique. Int. Orthod..

[22] Fillion D. (2011). Lingual straightwire treatment with the Orapix system. J. Clin. Orthod..

[23] Klocke A., Tadic D., Vaziri F., Kahl-Nieke B. (2004). Custom base preaging in indirect bonding. Angle Orthod..

[24] Muguruma T., Yasuda Y., Iijima M., Kohda N., Mizoguchi I. (2010). Force and amount of resin composite paste used in direct and indirect bonding. Angle Orthod..

[25] Park J.H., Choi J.Y., Kim S.H., Kim S.J., Lee K.J., Nelson G. (2021). Three-dimensional evaluation of the transfer accuracy of a bracket jig fabricated using computer-aided design and manufacturing to the anterior dentition: An in vitro study. Korean J. Orthod..

[26] Park K.H., Choi J.Y., Kim K.A., Kim S.J., Chung K.R., Kim S.H. (2021). Critical issues concerning biocreative strategy in contemporary temporary skeletal anchorage device orthodontics: A narrative review. Orthod. Craniofac. Res..

[27] Ludwig B., Alexander J.C., Cacciafesta V., Fillion D., Gilbert A., Moles R.C., Paz M.E., Silli S.M., Takemoto K. (2012). JCO roundtable. Lingual orthodontics. Part 1. J. Clin. Orthod..

[28] Spanou A., Koletsi D., Fleming P.S., Polychronopoulou A., Pandis N. (2016). Statistical analysis in orthodontic journals: Are we ignoring confounding?. Eur. J. Orthod..

[29] Goracci C., Özcan M., Franchi L., Di Bello G., Louca C., Vichi A. (2019). Bracket bonding to polymethylmethacrylate-based materials for computer-aided design/manufacture of temporary restorations: Influence of mechanical treatment and chemical treatment with universal adhesives. Korean J. Orthod..

[30] Kim D.Y., Ha S.W., Cho I.S., Yang I.H., Baek S.H. (2019). In-vitro investigation of the mechanical friction properties of a computer-aided design and computer-aided manufacturing lingual bracket system under diverse tooth displacement condition. Korean J. Orthod..

[31] Czolgosz I., Cattaneo P.M., Cornelis M.A. (2021). Computer-aided indirect bonding versus traditional direct bonding of orthodontic brackets: Bonding time, immediate bonding failures, and cost-minimization. A randomized controlled trial. Eur. J. Orthod..

[32] Schmid J., Brenner D., Recheis W., Hofer-Picout P., Brenner M., Crismani A.G. (2018). Transfer accuracy of two indirect bonding techniques—An in vitro study with 3D scanned models. Eur. J. Orthod..

[33] Duarte M.E.A., Gribel B.F., Spitz A., Artese F., Miguel J.A.M. (2020). Reproducibility of digital indirect bonding technique using three-dimensional (3D) models and 3D-printed transfer trays. Angle Orthod..

[34] Jackers N., Maes N., Lambert F., Albert A., Charavet C. (2021). Standard vs computer-aided design/computer-aided manufacturing customized self-ligating systems using indirect bonding with both. Angle Orthod..

[35] Niu Y., Zeng Y., Zhang Z., Xu W., Xiao L. (2021). Comparison of the transfer accuracy of two digital indirect bonding trays for labial bracket bonding. Angle Orthod..

[36] Wan Hassan W.N., Yusoff Y., Mardi N.A. (2017). Comparison of reconstructed rapid prototyping models produced by 3-dimensional printing and conventional stone models with different degrees of crowding. Am. J. Orthod. Dentofac. Orthop..

[37] Hazeveld A., Huddleston Slater J.J., Ren Y. (2014). Accuracy and reproducibility of dental replica models reconstructed by different rapid prototyping techniques. Am. J. Orthod. Dentofac. Orthop..

[38] Gyllenhaal K.A. (2015). Accuracy of Two Indirect Bonding Transfer Methods—A Three-Dimensional, In-Vivo Analysis. Master’s Thesis.

[39] Koch P.J. (2020). Measuring the accuracy of a computer-aided design and computer-aided manufacturing-based indirect bonding tray. Am. J. Orthod. Dentofac. Orthop..

